# A Perforated Mitral Valve Aneurysm: A Rare but Serious Complication of Aortic Valve Endocarditis Resulting From a Regurgitant Jet Lesion

**DOI:** 10.7759/cureus.11644

**Published:** 2020-11-23

**Authors:** Marije E Werner, Robert K Riezebos, Remko S Kuipers

**Affiliations:** 1 Cardiology, Onze Lieve Vrouwe Gasthuis, Amsterdam, NLD

**Keywords:** viridian's group endocarditis, mitral valve leaflet aneurysm, jet lesion, aortic valve endocarditis, septic embolism

## Abstract

Infective endocarditis has high morbidity and mortality rates. The aortic valve is most often affected in native valve endocarditis. Complications of aortic valve endocarditis range from local abscess and fistula formation, systemic complications secondary to thromboembolism and septic embolization, to congestive heart failure resulting from conduction system involvement and valve damage. A rare complication of aortic valve endocarditis is the occurrence of a ‘jet lesion’ on the mitral valve. Such a lesion, caused by an impinging regurgitant jet stream from a damaged aortic valve, can become directly and indirectly inoculated and evolve into a local infected aneurysm which might eventually rupture causing acute severe congestive heart failure and/or peripheral thromboembolism. We present the case of a 63-year-old man who presented with aortic valve endocarditis complicated by a perforated mitral valve aneurysm, congestive heart failure, and peripheral thromboembolism.

## Introduction

Infective endocarditis (IE) is the infection of the endocardial surface of the heart, mostly affecting native heart valves, but also affecting prosthetic material, such as artificial valves, conduits, patches, pacemakers, cardioverter-defibrillators, and catheters. Complications of valvular IE range from local abscess formation, fistula formation to the cardiac chambers, systemic complications secondary to thromboembolism and septic (mycotic) embolization, to congestive heart failure resulting from involvement of the conduction system and valvular destruction. Mitral valve aneurysm (MVA) formation, described as early as 1729 by Morand [1], is a rare complication of IE of the aortic valve. The incidence of MVA in the setting of IE has decreased from about 3.5% around 1970 [2] to less than 0.3% in more recent observational studies [3,4]. Despite diagnostic and therapeutic advances, IE remains a life-threatening disease associated with significant morbidity and mortality. Presentation is diverse, varying from patients presenting with isolated fevers and decompensated heart failure to patients presenting with hemorrhagic or ischemic stroke resulting from either septic or thrombus embolization [5]. Because of its many complications, early diagnosis and treatment of IE are of utmost importance [6]. We describe a case where delayed recognition of IE resulted in a rare but severe complication culminating in the death of our patient.

## Case presentation

We report the case of a 63-year-old male with a history of chronic pulmonary obstructive disease (COPD), atrial fibrillation, peripheral arterial vascular disease, and aortic regurgitation, due to an ascending aortic aneurysm and annular dilation for which a valve-sparing aortic root replacement (David procedure) had been performed 12 years previously. His medication included rivaroxaban, metoprolol, digoxin, furosemide, lisinopril, and salmeterol inhalers for his COPD. A complete overview of his medication at the time of presentation is listed in Table 1. One week prior to the current presentation he had been admitted to our emergency department (ED) with severe pain in his right arm. Upon admittance by an emergency physician there were no signs of fever. His pulse was irregular and the rate was within normal ranges. His blood pressure was 144/89 mmHg. Saturation was 97%. Auscultation of heart and lungs was not documented. Also, there was no documentation on the presence of endocarditis stigmata. Laboratory investigations showed elevated inflammatory parameters (see Table 2). An electrocardiogram (ECG) was not performed. No blood cultures were taken. After a CT-scan had revealed a thrombus in his right brachial artery the care of the patient was swiftly transferred to a vascular surgeon. Surgical embolectomy was performed within short notice. Elevated inflammatory parameters were attributed to ischemia in his arm and he was discharged the next day. A few days after discharge, it was realized that a source for his peripheral embolus had not been identified and our cardiology department was contacted to exclude a cardiac origin of the thrombus. We observed two minor Dukes criteria, i.e. the presence of an aortic graft after a David procedure and an arterial embolus. Because the patient had already been discharged he was contacted for further analysis. Since he was already on rivaroxaban for persistent atrial fibrillation and he reported no further complaints (e.g. no dyspnea or fevers), it was decided to perform ambulatory transthoracic echocardiography, possibly followed by transesophageal echocardiography, within two to three days to exclude a cardiac origin for his arterial embolus, such as infective endocarditis, left ventricular or left atrial appendage thrombus.

**Table 1:** Medication at the time of presentation

Medication	Dosage
Rivaroxaban	20 mg QD
Metoprolol ZOC	100 mg QD
Digoxin	0.125mg QD
Lisinopril	20 mg QD
Hydrochlorothiazide	12.5 mg QD
Metformin	1000 mg BD
Simvastatin	40 mg QD
Pantoprazol	40 mg QD
Tiotroprium inhaler	13 UG /inh QD
Salmeterol/fluticasone	50/500mcg/do BD

**Table 2:** Selection of Laboratory results at first presentation and at the second presentation BSE: blood sedimentation rate of erythrocyte, CRP: C-reactive protein, Nt-ProBNP: N-terminal (NT)-prohormone B-type natriuretic peptide, pCO2: partial pressure of carbon dioxide, pO2: partial pressure of oxygen

	1st presentation	2nd presentation
BSE, mm/hour	113	124
CRP, mg/L	121	131
Hemoglobin, mmol/L	7.8	6.7
Leucocytes, x10^9/l	12.9	13.7
Thrombocytes, x10^9/l	331	441
Nt-ProBNP, pmol/L	n/a	501
pH (7.35-7.45)	n/a	7.14
pCO2 (36-44 mmHg)	n/a	63
pO2 (70-100 mmHg)	n/a	29
Bicarbonate (22,0-29,0 mmmol/L)	n/a	21.5
Base Excess (-3.0-3.0 mmol/L)	n/a	-8.1
O2 saturation (95.0-98.0 %)	n/a	69.1
Lactate (0.5-1.7 mmol/L)	n/a	6.6

**Figure 1:**
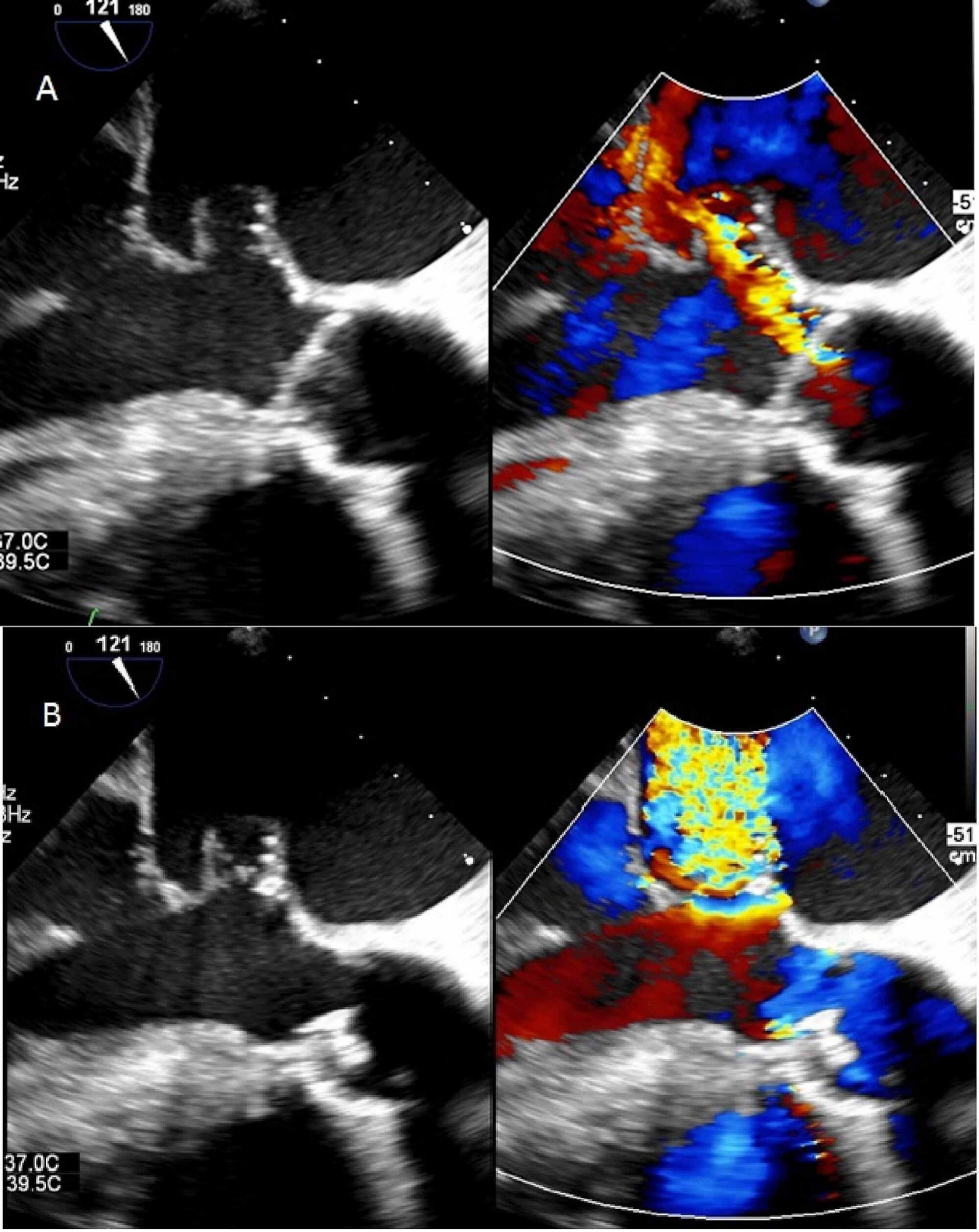
A: Aortic regurgitation with jet impinging on the anterior mitral valve leaflet. B: perforated aneurysm on the anterior mitral valve leaflet, leading to severe mitral regurgitation

Before these additional investigations were performed, the patient was re-admitted with sudden dyspnea. Physical examination showed desaturations down to 89%, but no fever. His blood pressure was 136/83 mmHg. Cardiac murmurs, pulmonary rales, and endocarditis stigmata were absent. Laboratory testing revealed elevated inflammatory markers and n-terminal-prohormone B-type natriuretic peptide (nt-ProBNP), and no signs of cardiac ischemia. Arterial blood gas analysis showed a respiratory acidosis and elevated lactate (see Table 2). An ECG showed atrial fibrillation with a ventricular rate of 110 bpm with a known left bundle branch block. Chest radiography showed consolidative abnormalities and pulmonary venous congestion. With a working diagnosis of pneumosepsis, with concomitant exacerbation of his COPD with secondary decompensated heart failure the patient was admitted to the pulmonology ward for treatment with amoxicillin and steroids. Blood cultures were taken. Within 24 hours, his condition deteriorated. He experienced severe dyspnea and he desaturated again to 88% while he was on oxygen therapy. His blood pressure was 155/92. He was transferred to the intensive care unit (ICU) for intubation, where he was stabilized. The antibiotic treatment regimen was changed to cefotaxime, ciprofloxacin in addition to steroids and diuretics. Transoesophageal echocardiography (TEE) was performed the next day. TEE revealed a 3 mm vegetation at the non-coronary cusp of the aortic valve (AV), indicative of IE, and a new, eccentric AV-regurgitation jet towards the anterior mitral valve leaflet (AMVL) (Figure 1A, Video 1). At the impact site of this aortic regurgitation jet, the AMVL showed severe regurgitation through a perforated aneurysm (Figure 1B, Video 1). This same day, the blood cultures revealed growth of *Streptococcus salivarius*, after which the dosage of the cefotaxime was raised to 12,000 mg/24h. Emergency cardiac surgery was scheduled but before it was started the patient developed signs of thrombo-embolic neurological complications. A CT-scan confirmed multiple extensive cerebral infarctions. Due to a now extremely poor prognosis palliation was initiated. The patient deceased the next day.

**Video 1:** perforated mitral valve aneurysm: a rare complication of aortic valve endocarditis resulting from a regurgitant jet lesion

In summary, we present the case of a patient that was initially admitted with a peripheral arterial embolism without a raised suspicion of IE, despite the presence of a predisposing heart condition. He was discharged after surgical embolectomy. Upon second presentation with congestive heart failure a ruptured AMVL with severe mitral regurgitation was revealed upon echocardiography. Before surgery could be performed the patient succumbed as a result of an embolic stroke.

## Discussion

**Figure 2:**
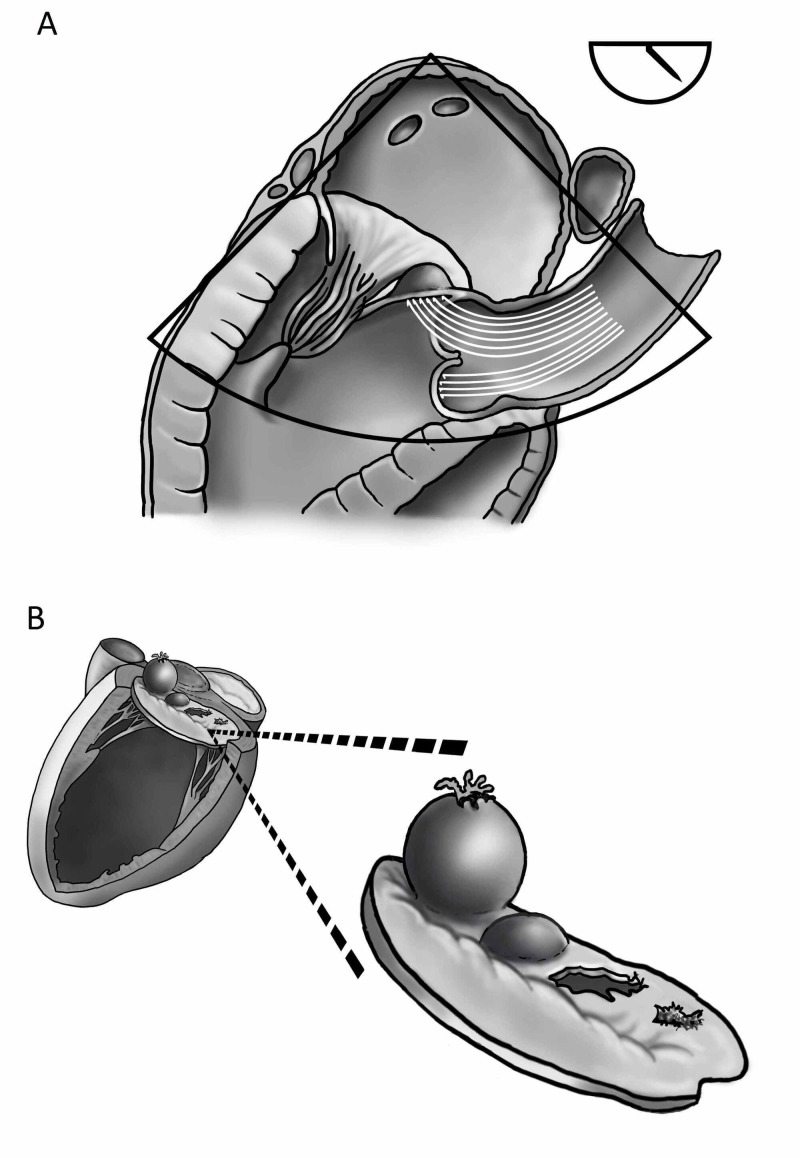
A: Mechanism of anterior mitral valve leaflet damage ( “ jet lesion” ) due to aortic valve regurgitation. The aortic valve (AV)-regurgitation jet impinges on the anterior mitral valve leaflet (AMVL), damaging the mitral valve apparatus. B: Variety of possible forms of damage to the anterior mitral valve leaflet due to jet lesions. From left to right: perforated aneurysm, aneurysm, perforation or rupture, endothelial damage.

Native aortic valve IE is the most predominant form of native valve endocarditis. Aortic valve regurgitation is a well-known complication of IE, but an endothelial lesion on the mitral valve apparatus secondary to an impinging jet originating from a destructed aortic valve is rare. This phenomenon is known as a ‘jet lesion’ (Figure 2A, Video 1). In the case of aortic valve IE this jet lesion may lead to secondary destruction of the mitral valve apparatus due to 1) the damaging effects of the jet on the endothelial surface of the valve itself and 2) through retrograde dissemination of bacteria [2,7]. Retrograde dissemination, in turn, might result from either 2A) direct contact of the aortic vegetation with the AMVL when aortic vegetations prolapse into the left ventricular outflow tract during diastole, so-called ‘mitral kissing vegetations,’ notably when the vegetation exceeds 6 mm in length, 2B) from secondary infection of the damaged endothelium by bacteria from the regurgitant blood flow and finally, retrograde dissemination can occur through 2C) local spread of the infection through the mitral-aortic intervalvular fibrosa [2,7]. As noted earlier, MVA occurs in about 0.3% of cases of IE at present [3,4]. Perforation of an MVA (Figure 2B, Video 1) seems to occur in about two-thirds of cases [8]. Interestingly, the size of the aneurysm does not seem to correlate with the risk of perforation [3]. The anterior leaflet is much more commonly involved than the posterior leaflet, for reasons that remain debated [3,4,9], but include its proximity to the aortic valve (see Figure 2A) and its more extensive vascularization [10]. In one cohort study of 211 patients with left-sided IE, *Streptococcus* species were shown to be the most common microorganism implicated in peri annular destruction [11].

The diagnosis of an MVA may be accomplished with transthoracic echocardiography but transesophageal echocardiography is more sensitive [9]. The echocardiographic appearance of MVA is a saccular bulge of the mitral leaflets, protruding into the left atrium during systole and subsequently collapsing during diastole [12]. For pre-operative investigation of the anatomical features of an MVA, real-time 3-dimensional echocardiography has been advised [13,14]. IE is the most predominant cause of MVA. Other conditions associated with MVA include connective tissue diseases (e.g. Marfan, Ehlers-Danlos, pseudoxanthomata elastic, and osteogenesis imperfecta), myxomatous valve degeneration, Libman-Sacks endocarditis, extreme forms of mitral valve prolapse, and congenital structural defects [9,12,15]. The differential diagnosis of MVA includes mitral valve diverticulum, blood cysts of the papillary muscle, cardiac masses (e.g. atrial myxoma of the mitral valve and papillary fibro-elastoma), chordal rupture, non-bacterial thrombotic endocarditis, (extreme) mitral valve prolapse, flail mitral leaflets, myxomatous degeneration, and infective vegetations. Vice versa MVA may be misdiagnosed as such [3]. A correct diagnosis is supported by the use of color flow doppler. Direct communication between the aneurysm and the left ventricle and a high-velocity regurgitant jet (Figure 1) supports the diagnosis of a perforated aneurysm [9].

Complications of an MVA include heart failure secondary to mitral valve regurgitation and thromboembolism. Of notice, the mitral valve may become regurgitant both due to a leaflet coaptation defect that results from the mass effect of the aneurysm, and from perforation of the aneurysm [9]. Thrombus may be present in the aneurysm [16] and aneurysm perforation might thus coincide with peripheral embolization. A recent review on aortic valve endocarditis complicated by MVA formation concluded that peripheral embolization is present in about 18% of cases in which perforation of the aneurysm is observed [8]. A significantly lower rate of embolization was observed when early surgery (<48h) was performed (21% vs 3%) [8]. Management of an MVA secondary to IE is mainly directed by the applicable cardiovascular guideline. As for infective endocarditis, the main arguments for surgery are refractory heart failure, uncontrolled infection, and the prevention of embolization [17]. When IE has been satisfactorily treated with antibiotics, the clinical condition of the patient, together with the severity of valve destruction and the presence or absence of perforation determine the indication for intervention [18]. A recent review of about 30 published cases of MVA associated with IE showed that surgery is performed in about 75% of cases [8]. In some cases, with a favorable clinical presentation, rapid control of infection with antibiotics and the absence of other guideline-recommended reasons for surgery, conservative treatment can be initiated under close clinical and echocardiographic monitoring [19].

Finally, the present case illustrates another challenge the physician faces, since the diagnosis of IE was clearly missed initially during the first visit of the patient to the hospital. As stated in the latest 2015 European Society of Cardiology (ESC) guideline on IE, IE should be suspected in a variety of very different clinical situations [17]. However, except for the combination of fever and embolic phenomena, this guideline does not provide clear advice on when IE should be suspected with regard to e.g. the cumulative number of Dukes criteria. Moreover, in many countries a patient with a peripheral embolism is presented at an ED and is subsequently seen by an emergency physician who contacts a second specialist, in our case a vascular surgeon. As illustrated by the present case, a cardiologist should be, but is not necessarily, contacted despite the presence of various Dukes criteria. Moreover, the treating physician may well be unaware of the content of cardiology guidelines or the various (modified) Dukes criteria for IE. In the present case, a diagnosis of IE had not at all been suspected due to the absence of fever and the fact that the elevated markers of inflammation could also be explained by ischemia due to the embolus in the brachial artery. On the other hand, the addition of one major Dukes criterium (i.e. evidence of endocardial involvement by echocardiography or a positive blood culture) to the already present two minor criteria (i.e. a predisposing heart condition - an aortic graft after a David procedure- and a peripheral arterial embolism) would have changed the diagnostic result for IE from ‘rejected’ to ‘possible’ according to the Dukes algorithm. We suggest that, since not all physicians are familiar with the differential diagnosis of a peripheral arterial embolism from a cardiovascular point of view, physicians should be trained to be aware of the Dukes criteria for IE. IE should be suspected and thus excluded by additional investigations when at least one major or more than one minor Dukes criterium is present. Hence, when a patient is admitted with at least one major or more than one minor Dukes criterium the other criteria should proactively be investigated. In our case, endocarditis stigmata should have been looked for and recorded. The cardiologist should have been contacted at an early stage. In the present case, this would likely have resulted in the detection of IE and possibly the presence of an MVA before rupture.

## Conclusions

Secondary mitral valve involvement in aortic valve endocarditis is a rare and severe complication with, notably in case of a ruptured MVA, relatively high rates of congestive heart failure and peripheral embolization. In case of jet lesions of the mitral valve secondary to aortic valve endocarditis, early surgery of the aortic valve can preserve mitral valve integrity and therefore improve prognosis. We want to emphasize that in cases of a low suspicion of IE (i.e. one major or more than one minor Dukes criterium), and more specific, in cases with a predisposing heart condition, additional investigation of the other Dukes criteria, should be performed to exclude the life-threatening complications of IE, such as a (perforated) MVA.
